# Screening and brief intervention for alcohol problems in Dr George Mukhari Hospital out-patients in Gauteng, South Africa: a single-blinded randomized controlled trial protocol

**DOI:** 10.1186/1471-2458-12-127

**Published:** 2012-02-14

**Authors:** Supa Pengpid, Karl Peltzer, Linda Skaal, Hendry van der Heever, Guido Van Hal

**Affiliations:** 1 Department of Health System Management and Policy, University of Limpopo (MEDUNSA Campus), Pretoria, South Africa; 2 HIV/AIDS/STI and TB (HAST) Research Programme, Human Sciences Research Council, Pretoria, South Africa; 3 Department of Psychology, University of Limpopo, Turfloop, South Africa; 4 University Scientific Institute for Drug Problems, University of Antwerp, Antwerp, Belgium

## Abstract

**Background:**

For alcohol drinkers in South Africa it has been found that annual consumption per drinker is among the highest in the world. High prevalence rates of hazardous and harmful alcohol use have also been found in a hospital out-patient setting in South Africa. Hospital settings are a particularly valuable point of contact for the delivery of brief interventions because of the large access to patient populations each year. With this in mind, the primary purpose of this randomized controlled trial is to provide screening for alcohol misuse and to test the efficacy of brief interventions in reducing alcohol intake among hospital out-patients in South Africa.

**Methods/Design:**

The study design for this efficacy study is a randomised controlled trial with 6- and 12-month follow-ups to examine the effects of a brief alcohol intervention to reduce alcohol use by problem drinkers in a hospital setting. The unit of randomisation is the individual out-patient identified as a medium risk drinker attending Dr George Mukhari Hospital. Out-patients will be screened for alcohol problems, and those identified as medium risk drinkers will be randomized into an experimental or control group. The experimental group will receive one brief counselling session on alcohol risk reduction, while the control group will receive a health education leaflet.

**Discussion:**

The trial will evaluate the impact of alcohol screening and brief interventions for patients with alcohol problems in a hospital out-patient setting in South Africa. The findings will impact public health and will enable the health ministry to formulate policy related to brief alcohol interventions, which will result in reduction in alcohol use.

**Trial registration:**

PACTR201110000319392

## Background

Of the 20 countries in Africa identified by the World Health Organisation (WHO) with very high levels of child and adult mortality, it was estimated that for 2000 the total alcohol consumption per adult was 7.1 litres of absolute alcohol [1]. However, when one takes into consideration that a relatively low percentage of adults in these countries including South Africa consume alcohol (55% of men and 30% of women), the annual consumption per drinker increases to 16.6 litres of absolute alcohol, the highest level for any region of the world [1]. In addition, the overall pattern of drinking in the African region in 2000 was ranked as the second most detrimental in the world (indicative of high rates of binge drinking and alcohol dependence) with 1% of all deaths among women and 4% of all deaths among men attributable to alcohol [1,2]. Corresponding figures from South Africa were 10.7% for men and 3.1% for women [3].

In a sample of 1532 (56.4% men and women 43.6%) hospital outpatients in South Africa, 41.2% of men and 18.3% of women were found to be hazardous drinkers, and 3.6% of men and 1.4% of women meet criteria for probable alcohol dependence or harmful drinking as defined by the Alcohol Use Disorder Identification Test (AUDIT) [4]. Strebel, Stacey and Msomi [5] investigated psychiatric hospital patient records (n = 7938) in Cape Town and found that alcohol abuse was prevalent among 6.3% of women and 15.1% of men. Hospital settings are a particularly valuable point of contact for the delivery of brief interventions because of the large access to patient populations each year [6,7]. A number of randomised controlled trials have shown [8] including more recently two trials in non-health care settings in low and middle income countries [9,10] that, in comparison with controls, hazardous and harmful drinkers receiving 5-10 minutes of brief structured advice plus a self-help booklet from health care workers will reduce alcohol consumption by an average of 25%. Overall, it has been estimated that around 20% of patients identified as hazardous or harmful drinkers who receive a brief intervention will reduce their alcohol consumption [11].

The proposed project is a randomized controlled trial aimed at evaluating Screening and Brief Intervention (SBI) for alcohol problems among hospital out-patients in Gauteng province, South Africa.

### Aim of the study

The aim of this study is to assess the effectiveness of Screening and Brief Intervention (SBI) for alcohol problems among hospital out-patients in South Africa using a randomized controlled trial design.

### Objectives

1. To measure the prevalence of alcohol consumption amongst out-patients at Dr George Mukhari Hospital.

2. To describe drinking patterns of out-patients at Dr George Mukhari Hospital, and identify medium risk drinkers needing intervention.

3. To compare level of alcohol consumption amongst medium risk drinkers between pre-intervention, 6 months, and 12 months after intervention among out-patients at Dr George Mukhari Hospital.

4. To compare alcohol consumption between intervention and control groups at 6 and 12 months.

## Methods/Design

### Design

The study design for this efficacy study is a randomised controlled trial with 6- and 12-month follow-ups to examine the effects of a brief alcohol intervention to reduce alcohol use by problem drinkers in a hospital setting. The unit of randomisation is the individual out-patient identified as a medium risk drinker attending Dr George Mukhari Hospital.

### Study population and participants

The sample will include out-patients of Dr George Mukhari Hospital. Out-patients will be screened for alcohol problems, and those identified as medium risk drinkers will be randomized into an experimental or control group. The experimental group will receive one brief counselling session on alcohol risk reduction, while the control group will receive a health education leaflet. The control group will be offered a delayed brief alcohol intervention if the intervention proves to be efficacious.

#### Study hypotheses

• Out-patient medium risk drinkers in the intervention group reduce drinking much more than those in the control group.

• The drinking patterns of patients in the intervention group will continuously reduce over a 12 month assessment period.

### Principles for recruitment

#### Inclusion criteria

Out-patients (males and females) 18 years and above, without mental impairment, who visit the hospital out-patient department and who scored as medium risk drinkers (i.e. 8-19 for men and 7-19 for women on the AUDIT questionnaire) will be included in this study.

#### Exclusion criteria

Out-patients with a score of 20 and above on the AUDIT (with probable alcohol dependence). Also, out-patients who score less than 8 for men and less than 7 for women on the AUDIT questionnaire, patients with mental impairment, those who are pregnant, and those who are already under alcohol treatment, will all be excluded.

### Randomization

After baseline assessment, each patient is randomized to either a control or a brief intervention group. Randomisation is achieved by concealed centrally-allocated computer generated random numbers.

### Blinding

Hospital staff members and out-patients will not be blind to their intervention or delayed intervention status. However, to protect against information biases in the reporting of alcohol use behaviour, the data collection team who will assess the outcomes will be blind to the client's status as intervention or delayed intervention arm.

### Procedure

**Figure 1:**
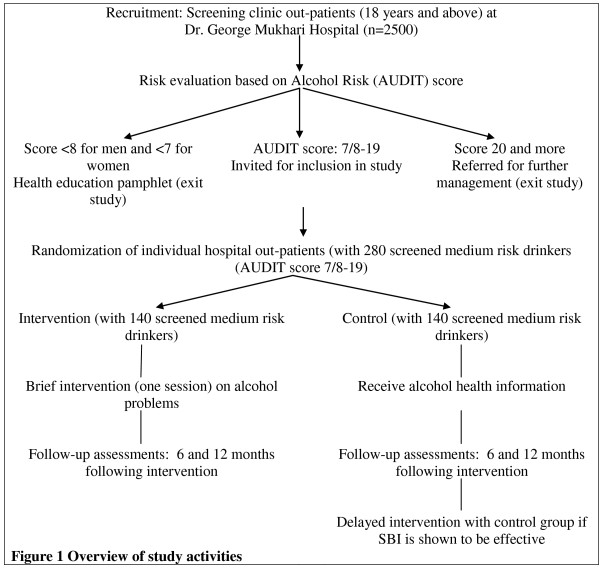
**Overview of study activities**.

Universal screening of all presenting out-patients will be used whereby all consecutive clients visiting out-patient departments will be screened for alcohol problems and randomized into an intervention or control group. Research assistant 1 will ask for consent from patients attending the hospital out-patient department to participate in the study, i.e. do a baseline assessment using the AUDIT questionnaire. Research assistant 1 will not be involved in delivering treatment. Research assistant 2 will score the results of the alcohol test section of the questionnaire. Hospital out-patients who score 8-19 for men and 7-19 for women on the AUDIT questionnaire after screening (risky drinkers) will be included in this study. Patients with a score of 20 and above on the AUDIT (with probable alcohol dependence) will be referred for further management. Research assistant 2 implements the randomization to intervention or control arms. Research assistant 2 will carry out the intervention for all the participants, after which they will be followed up at 6 months and 12 months, and assessments will be done by Research assistant 1, who will be blinded to the intervention allocation of the participants. In the event of a drop-out, at least six individual attempts will be made to contact patients by telephone and letter. Even if a contact was not successful at 6 months, further attempts will be made at 12 months. Sampling will occur throughout all hours of clinic operation over a 4-month period. Two-hundred and eighty patients will be recruited from hospital out-patient departments. They will receive 40 South African Rands for transport for returning to the hospital and completing each of the two follow-up assessments (in total R 80). (see Figure 1)

#### Consent

Consent to participate will be obtained in a 2-stage process. Research assistants will initially ask for informed consent to conduct health screening and collect some basic demographic information and check eligibility to take part. No identifiable information will be collected at this stage. Patients who then are positive on the AUDIT (alcohol medium risk score), as applicable, will have the study explained to them verbally by another research assistant counsellor and in writing (using the patient information leaflet). Informed consent will be obtained at this second stage which will include permission to give the patient's data and contact details to the research staff, and participate in the experimental or control condition and follow up after 6 and 12 months by the research assistant.

### Interventions

#### Control arm

Participants randomized to this group will not receive feedback on the initial alcohol screening. They will be provided with a health education leaflet on responsible drinking, and they will only receive the brief intervention at the 12 month follow-up if the intervention has proved to be efficacious.

### Experimental arm: brief intervention

Participants who are randomized onto the brief intervention arm receive personalized feedback on their AUDIT results, a health education leaflet, simple advice plus brief counselling about reducing excessive drinking, during a one session 20 minute intervention. The steps of brief counselling are: 1) To identify any alcohol-related problems mentioned in the interview, 2) To introduce the sensible drinking leaflet, emphasise the idea of sensible limits, and make sure that patients realize that they are in the medium-risk drinking category, 3) To work through the first 3 sections of the problem solving manual while mentioning the value of reviewing the other sections, 4) To describe drinking diary cards, 5) To identify a helper, and 6) To mention the 6 and 12 months follow-up assessments.

The Information-Motivation-Behavioural Skills (IMB) Model will be used to guide the alcohol reduction intervention. The IMB model [12-14] proposes that *information *about alcohol misuse and methods of reducing and preventing harmful and/or hazardous drinking is a necessary precursor to risk reduction. *Motivation *to change, however, also directly affects whether one acts on information about risk and risk reduction. Finally, the IMB model holds that *behavioural skills *related to preventive actions represent a final common pathway for information and motivation to result in alcohol risk behaviour change. The IMB model posits that information and motivation activate behavioural skills to ultimately enact risk reduction behaviours. The IMB model also shows that information or motivation alone can have direct effects on some preventive behaviours, such as when information about risky alcohol drinking prompts drinking in moderate levels or to stop drinking.

### Counsellor training and intervention quality assurance

The intervention research assistant counsellor will deliver the interventions to men and women patients as per usual clinic services. There is a comprehensive manual for the brief intervention which will be used to guide the research assistant counsellor throughout the session. The research assistant counsellors will be trained to administer the intervention protocol through role playing and general skills training techniques in a 5 day workshop. Site visits will be done bi-weekly by the project manager to offer support and supervision to the trained research assistant counsellors. In terms of control around the quality and consistency of the implementation of the intervention, research assistants will indicate which intervention was provided to the patient as part of an addition to the AUDIT questionnaire. In addition, research assistants will be able to report to their coordinators regarding any problems they may be having in implementing the brief intervention. Regular meetings between the researchers and the project manager will allow for any problems to be resolved in good time. Prior to intervention implementation, research assistants will be observed in role-play demonstrations until performance criteria are met. In addition, during implementation, research assistants will be observed "in vivo" for adherence to the protocol. Weekly supervision will be provided by a trained counsellor mentor.

#### Outcome measures

##### Demographic characteristics

A researcher-designed questionnaire will be used to record information on participants' age, gender, educational level, marital status, income, and residential status.

##### Health-status

The SF-12 is a multipurpose short-form (SF) generic measure of health status. The SF-12 Health Survey includes 12 questions from the SF-36 [15]. These include: 2 questions concerning physical functioning; 2 questions on role limitations because of physical health problems; 1 question on bodily pain; 1 question on general health perceptions; 1 question on vitality (energy/fatigue); 1 question on social functioning; 2 questions on role limitations because of emotional problems; and 2 questions on general mental health (psychological distress and psychological well-being). In addition, participants will be asked about a list of chronic conditions such as heart disease and diabetes.

##### Alcohol consumption

The 10-item Alcohol Disorder Identification Test (AUDIT) [16] assesses alcohol consumption level (3 items), symptoms of alcohol dependence (3 items), and problems associated with alcohol use (4 items). Responses to items on the AUDIT are rated on a 4-point Likert scale from 0 to 4, with a maximum score of 40 points. AUDIT scores higher than 19 indicate more severe levels of risk; scores of 8-19 in men and 7-19 in women indicate a tendency to problematic drinking. To comply with the timeline of this study, all subjects will be asked for their alcohol consumption in the previous 6 months rather than 1 year.

The primary outcome measure used in this study is the change in drinks per week from baseline to follow-up, as estimated from the first two AUDIT questions. A secondary measure is the frequency of consuming four or more drinks per occasion (AUDIT question 3). The percentage of patients whose weekly consumption decreases, increases, or remains the same, as well as changes in a combination of the first three AUDIT item scores called the Drinkers' index, will also be evaluated in order to estimate the amount of alcohol risk reduction.

##### Tobacco use

Two questions will be asked about the use of tobacco products.

Heath status and tobacco use questions are integrated into the screening of alcohol use so as to reduce possible stigma of reporting alcohol use.

This questionnaire will be administered at baseline, 6 and 12 months follow-up visits. All questionnaires will be administered in English or Tswana, the two languages predominantly spoken by nearly all clinic patients.

### Sample size calculation

The sample size was calculated using the Protocol Support Tool for Randomize Control Trial Research Software (PST Version 1), for a two arm randomized controlled trial, with 80% power, significance level of 5%, two sided-tests, standard deviation of the control group measurement of 5, and a mean difference between intervention and control of 2. The minimum sample size of each group is 99. To accommodate a drop-out rate of 15% at each follow up, the sample size for each group has been increased to 140, this makes 280 in total.

### Data analysis

Means, standard deviations, and percentages will be used for descriptive statistics. *T*-test for continuous data and chi-square for categorical data will be used to examine differences between groups. Generalized Linear Model Repeated Measures 2 × 3 Analysis of Variance (ANOVA) will be used for comparing observations (alcohol use score) across the three contact periods to demonstrate a treatment intervention × time interaction. Observations with a single follow-up point missing (at either 6 or 12 months) will be imputed with the available follow-up. Data for participants who are lost to follow-up at both 6 and 12 months will be imputed using baseline values. SPSS for Windows version 17.0 (SPSS, Inc., Chicago, IL) will be used for calculations.

#### Ethical and governance approval

We have received ethical approval from the Medunsa Research and Ethics Committee (Project number: MREC/H/220/2010:IR). Dr George Mukhari Hospital has also provided approval for this study.

#### Project timescales

The study will run for a period of 16 months beginning in February 2011 to June 2012.

## Abbreviations

AUDIT: Alcohol Use Disorder Identification Test; IMB: Information-Motivation-Behavioural Skills; SBI: Screening and Brief Intervention; SF-12: A 12-Item Short-Form Health Survey

## Competing interests

The authors declare that they have no competing interests.

## Authors' contributions

SP and KP were the main contributors to the conceptualization of the study. SP and KP also contributed significantly to the first draft of the paper and all authors contributed to the subsequent drafts and finalization. All authors read and approved the final manuscript.

## Pre-publication history

The pre-publication history for this paper can be accessed here:

http://www.biomedcentral.com/1471-2458/12/127/prepub
